# Detachment Activated CyPA/CD147 Induces Cancer Stem Cell Potential in Non-stem Breast Cancer Cells

**DOI:** 10.3389/fcell.2020.543856

**Published:** 2020-10-16

**Authors:** Yao Meng, Xin-Yu Fan, Li-Jun Yang, Bao-Qing Xu, Duo He, Zhe Xu, Dong Wu, Bin Wang, Hong-Yong Cui, Shi-Jie Wang, Li-Juan Wang, Xiao-Qing Wu, Jian-Li Jiang, Liang Xu, Zhi-Nan Chen, Ling Li

**Affiliations:** ^1^National Translational Science Center for Molecular Medicine, Department of Cell Biology, School of Basic Medicine, The Fourth Military Medical University, Xi’an, China; ^2^Shaanxi Provincial Centre for Disease Control and Prevention, Xi’an, China; ^3^Department of Urology, Xijing Hospital, The Fourth Military Medical University, Xi’an, China; ^4^Department of Pathology, Fuzhou General Hospital of Nanjing Military Command, Fuzhou, China; ^5^Department of Molecular Biosciences, The University of Kansas, Lawrence, KS, United States; ^6^Department of Radiation Oncology, The University of Kansas, Lawrence, KS, United States

**Keywords:** breast cancer, detachment culture, induced cancer stem cells, CD147, CyPA

## Abstract

**Background:**

Cancer stem cells (CSCs), responsible for cancer metastasis and recurrence, are generated from non-CSCs after chemo-radiation therapy. This study investigated the induction of CSC potential in non-stem breast cancer cells and the underlying molecular mechanisms in detachment culture.

**Methods:**

Bulk breast cancer cells, or sorted non-CSCs and CSCs were cultured under an attached or detached condition to assess CSC numbers, ability to form tumor spheres, expression of stemness markers, and chemoresistance. Lentivirus carrying CD147 shRNA or cDNA was used to manipulate CD147 expression, while CD147 ligand recombinant cyclophilin A (CyPA) or its inhibitor was used to activate or inhibit CD147 signaling.

**Results:**

Detachment promoted anoikis resistance, chemoresistance, sphere formation, self-renewal, and expression of stemness markers in breast cancer cells. Detachment increased functional ALDH^+^ or CD44^high^CD24^–/*low*^ CSCs, and induced CSC potential in ALDH^–^ or CD44^*low*^CD24^high^ non-CSCs. Upon detachment, both CD147 expression and CyPA secretion were enhanced, and CyPA-CD147 activation mediated detachment induced CSC potential in non-CSCs *via* STAT3 signaling. Clinically, CD147 and pSTAT3 were highly co-expressed and correlated with poor overall survival and tumor recurrence in breast cancer patients.

**Conclusion:**

This study demonstrates that detachment induces the generation of CSCs from non-stem breast cancer cells *via* CyPA-CD147 signaling, indicating that targeting CD147 may serve as a potential novel therapeutic strategy for lethal metastatic breast cancer by eliminating induced CSCs.

## Introduction

Breast cancer is the most commonly diagnosed cancer in women and the sixth leading cause of cancer-related deaths in Chinese women (Li et al., 2016). Approximately 80% of breast cancer-related deaths are due to cancer metastasis to distant organs, like the lung, bone, liver, or brain (Quaglino et al., 2020). To date, surgery is still the most effective treatment option to cure localized breast cancer, and chemo-radiotherapy shows efficacy for shrinking tumors; however, both treatment strategies fail to eradicate tumor metastasis. Thus, there is an urgent unmet need to develop novel and more effective strategies to control breast cancer metastasis.

Cancer cells can spread in the early stage of disease, such as cancer *in situ* or precancerous lesions, while clinically visible cancer metastasis usually occurs over time, typically 5–20 years (Visvader and Lindeman, 2012). In an experimental mouse model, the majority of injected metastatic cancer cells were shown to undergo apoptosis within 2 days and only 0.02% of cancer cells were able to form macro-metastases (Oskarsson et al., 2014; Celia-Terrassa and Kang, 2016). It is estimated that millions of cancer cells are released from a primary tumor mass into the circulation system daily; however, only a few of these cells can survive and successfully form metastatic lesions. This small portion of cancer cells are referred to as cancer stem cells (CSCs), which possess the ability to initiate tumor formation and growth or to initiate tumor metastasis and recurrence in patients (Batlle and Clevers, 2017; Clarke, 2019). Numerous reports have provided evidence that CSCs exist in the blood and solid tumor, and breast CSCs were among the earliest reported CSCs isolated from solid tumors (Al-Hajj et al., 2003). Breast CSCs were found to have higher specific cell surface antigen CD44 expression but no or lower CD24 expression, or to have higher aldehyde dehydrogenase 1 (ALDH1) activity (Celia-Terrassa and Kang, 2016; Cortes-Hernandez et al., 2019). Besides their tumorigenicity capability, breast CSCs exhibit enhanced *in vitro* invasiveness and *in vivo* metastasis compared with non-CSCs; and high numbers of CSCs have been associated with poor prognosis and distant metastasis in breast cancer patients (Pece et al., 2010).

Recently, it has become commonly accepted that not all CSCs have an equal capability to initiate metastasis, and that only a subset of CSCs, named as metastatic stem cell (MetSC) (Oskarsson et al., 2014) or metastasis-initiating cells (MICs) (Celia-Terrassa and Kang, 2016), possesses the ability to settle metastatic colonies in secondary organs. Beside inherited CSC potential, including self-renewal, differentiation, and tumorigenicity, MICs may acquire additional properties, such as resistance to anoikis, which is a detachment induced apoptosis that serves as the first major challenge and a critical barrier to metastasis. These properties provide the cells with a chance to survive in the circulation and form metastasis (Peitzsch et al., 2017). Moreover, MICs may have evolved from a subpopulation of circulating tumor cells (CTCs, cancer cells detached from a solid tumor mass into the blood circulatory system) by undergoing epithelial to mesenchymal transition (EMT) and obtaining induced stem cell-like potential, which provides them an extraordinary advantage to suppress anoikis and seed a metastatic lesion (Pece et al., 2010; Gkountela and Aceto, 2016; Peitzsch et al., 2017). In breast cancer CTC populations, the existence of CD44^+^Met^+^CD47^+^ MICs has been reported (Pece et al., 2010).

Thus, MICs with both CSC potential and anoikis resistance properties are likely driven by epigenetic changes and subsequent induction under extreme stress conditions, like detachment from the extracellular matrix (ECM) during dissemination (Peitzsch et al., 2017). Hypomethylation regions enriched with transcription factor binding sites for stemness genes, such as OCT4, NANOG, and SOX2, were identified in metastasis-prone CTC clusters (Gkountela et al., 2019). However, it remains unclear how the CTC epigenetic status or the induced CSC potential is established under the ECM detached condition. Therefore, to discover a novel anti-metastasis strategy against MICs, research exploring the impact of detachment on CSC potential and the corresponding molecular mechanisms is urgently needed. Furthermore, current research in the field has mainly focused on enrichment and assessment of CSCs using the sphere formation assay in a detached or non-adherent condition, but only a few studies have assessed the induction of CSC potential upon detachment.

Our previous studies demonstrated that breast cancer cells with high CD147 expression possess more malignant phenotypes, like cancer metastasis and recurrence, and are associated with poor overall survival and treatment outcomes (Li et al., 2009). CD147 promotes cancer cell invasion and metastasis by stimulating the secretion of matrix metalloproteinase (Xu et al., 2007b), enhancing cell motility (Zhao et al., 2011), and regulating the interaction between cancer and stroma (Xu et al., 2013). More importantly, CD147 expression can induce cancer cells to acquire CSC characteristics, such as EMT (Wu et al., 2011), anoikis resistance (Ma et al., 2010; Ke et al., 2012), and chemo-resistance (Tang et al., 2012). In addition, we previously reported that CD147 induces cell proliferation and invasion by activating pSTAT3^Y705^ signaling *via* interacting with CD44s (Li et al., 2013), while anti-CD147 inhibits CSC potential and sensitizes cancer cells to chemoradiotherapy by blocking CD44s-pSTAT3 signaling (Fan et al., 2019). In view of the role of CD147 in CSCs and anoikis resistance, we hypothesized that CD147 may be involved in the induction of breast CSC features under a detached condition.

We performed a systematic study to explore the impact of detachment on the potential of breast CSCs and the underlying cellular and molecular mechanisms. Our findings provide a promising therapeutic strategy to prevent breast cancer metastasis through disrupting detachment induced CSC potential *via* targeting CD147.

## Materials and Methods

### Cell Lines and Constructs

Human breast cancer T-47D, MCF7, and MDA-MB-231 cell lines were originally purchased from the American Type Culture Collection (Manassas, VA, United States) and cultured in Roswell Park Memorial Institute-1640 medium (RPMI-1640; HyClone, Logan, UT, United States) supplemented with 10% fetal bovine serum (FBS, HyClone) in a humidified incubator with 5% CO_2_ at 37°C. All cell lines were tested for mycoplasma and characterized by short tandem repeat (STR) profiling analysis (Cenvino, Beijing, China).

The pGIPZ empty vector, pGIPZ-CD147, and pLKO.1-CD147 lentiviral shRNA constructs were obtained from Open Biosystems (Lafayette, CO, United States). The MISSION^®^ Non-Target shRNA control vector (pLKO.1-NTC) was obtained from Sigma Chemicals. CD147 overexpressed construct was established by subcloning human CD147 cDNA into the GV341 lentiviral expression vector (Genechem, Shanghai, China).

### Establishment of Stable Cell Sublines

The lentivirus carrying CD147 shRNA (untagged pLKO CD147-shRNA, GFP tagged pGIPZ CD147-shRNA) or negative control shRNA, carrying CD147 cDNA or control vector was infected into breast cancer cells, and the stable cell subclones were selected by adding culture medium containing 3–6 μg/ml of puromycin. CD147 expression levels were analyzed using flow cytometry and immunoblotting.

### Tumorsphere Culture and Passaging

Cells were suspended in Dulbecco’s modified Eagle’s medium/F12 (DMEM/F12, HyClone) serum-free medium containing 1% N2, 2% B27, 15% glucose (Invitrogen), 10 ng/ml human bFGF (Sigma Chemicals), 10 ng/ml EGF (Invitrogen), 1 μg/ml hydrocortisone (Sigma Chemicals), 5 μg/ml insulin (Sigma Chemicals), 5 μg/ml β-mercaptoethanol (Invitrogen), 0.2% heparin (Stem Cell Technologies, Cambridge, MA, United States) and seeded into six-well ultra-low attachment plates (Corning, NY, United States) in triplicate at a density of 2 × 10^4^ cells per well and cultured for 10–14 days to form tumor spheres, which were quantified under an inverted microscope (Olympus). For tumorsphere passaging, we collected spheroids with a 40 μm Cell Strainer (Corning, San Jose, CA, United States) and disassociated them with TrypLE^TM^ (Thermo Fisher Scientific, Waltham, MA, United States) into a single cell suspension, and re-seeded the viable cells into six-well ultra-low attachment plates at a density of 2 × 10^4^ cells per well and cultured for 10–14 days. Sphere formation efficiency (SFE) was calculated as the percentage of the number of spheres larger than 70 μm in the total number of original inoculated cells.

For single cell tumorsphere formation, cells were seeded into 96-well ultra-low attachment plates (Corning) at a dilution of approximately one cell per well and maintained for 1 month with the tumorsphere medium refreshed every 7 days.

### Anoikis Assay

Cells were seeded into 24-well ultra-low attachment plates (Corning, detached condition) or uncoated common plates (attached condition) at a density of 5 × 10^4^ per well and cultured in RPMI-1640 medium containing 10% FBS for indicated periods of time. The cells were labeled with the Annexin V-FITC and propidium iodide (PI) double staining kit (BioLegend, San Diego, CA, United States) according to the manufacturer’s protocol. The labeled cells were immediately measured with a BD FACS Calibur Flow Cytometer (BD Biosciences, San Jose, CA, United States) and quantified with CellQuest software (BD Biosciences) for apoptosis (anoikis) levels.

### ALDEFLUOR Assay and Sorting of ALDH^±^ Subpopulations

The ALDEFLUOR kit (Stem Cell Technologies) was applied to analyze the population with ALDH1 enzymatic activity according to a previous study (Wu et al., 2016). In brief, 1 × 10^6^ cells were incubated in the ALDEFLUOR assay buffer containing 1 μM ALDH1 substrate BAAA at 37°C for 45 min, whereas negative control cells were incubated with 50 mM of ALDH inhibitor diethylaminobenz aldehyde (DEAB) under the same conditions. Next, the cells were resuspended in 2% FBS/Hank’s Balanced Salt Solution (HBSS) containing 1 μg/ml 7-AAD to exclude dead cells. The sorting gates were established using DEAB negative cells, and positive and negative cells were sorted on a BD FACS Aria III (BD Biosciences). The purity of sorted cells was determined as >90% positive.

### CD44^+^CD24^–^ Staining and Sorting of CD44^high^CD24^–/*low*^ or CD44^*low*^CD24^high^ Subpopulations

1 × 10^6^ cells were incubated on ice with 200 μl of anti-CD24-PE and anti-CD44-APC in 2% FBS/HBSS in the dark for 20 min, and the antibody labeled cells were then analyzed using a FACS Calibur Flow Cytometer. The isotype-matched mouse immunoglobulin was used as a negative control. For cell sorting, cells were stained with the indicated antibodies and suspended in 2% FBS/HBSS with 1 μg/ml 7-AAD for gating viable cells. Positive and negative cells were sorted on a BD FACS Aria III. The purity of sorted cells was determined as >90% positive.

### Patient Samples and Tissue Microarray

A total 138 breast cancer patients who were hospitalized between June 2007 and August 2016 were included in this retrospective study. Patients’ survival data were recorded for 26–132 months. All patients (100%) underwent primary surgical interventions. The median age of patients was 54 years (range 29–87 years). Clinicopathological data were collected from patients’ medical records, which included age of patients at diagnosis, tumor type and grade, TNM stage, AJCC stage, patient survival, recurrence time, and the expression of Her-2, ER, PR, P53 (Table 1). This study was approved by the Institutional Review Board of the Air Force Medical University. A tissue microarray (TMA) constructed from paraffin-embedded tissue blocks from above 138 patients was purchased from the National Engineering Center for Biochip (Shanghai, China). The study protocol was approved by the ethics committee of The Fourth Military Medical University (KY20183305-1).

**TABLE 1:** Characteristics of breast cancer patients.

Clinical features	# Patients (%)
**Age, year**	
Mean, median (range)	55.7, 54 (29–87)
**Tumor type**^∗^	
IDC	125 (91.2)
ILC	3 (2.2)
Mucinous adenocarcinoma	6 (4.4)
Others	3 (2.2)
**Tumor grade**	
II	96 (69.6)
II–III	37 (26.8)
III	5 (3.6)
**T stage**	
T1	56 (40.6)
T2	80 (58.0)
T3	2 (1.4)
**N stage**	
N0	74 (53.6)
N1	18 (13.1)
N2	38 (27.5)
N3	8 (5.8)
**M stage**	
M0	127 (92.0)
M1	11 (8.0)
**AJCC clinical stage**	
I	33 (23.9)
IIA–IIB	59 (42.8)
IIIA–IIIC	46 (33.3)
**Overall survival, month**	
Mean, median (range)	108.9, 121 (26–132)
Live	109 (79.0)
Dead	29 (21.0)
**Recurrence, month**	
Mean, median (range)	100.7, 119 (10–132)
No	100 (72.5)
Yes (with metastasis)	38 (27.5) [11 (8.0)]
**Her-2**^#^	
Negative	62 (52.5)
Positive	56 (47.5)
**ER**^#^	
Negative	43 (31.9)
Positive	92 (68.1%)
**PR**^#^	
Negative	65 (47.4)
Positive	72 (52.6)
**P53**^#^	
Negative	66 (79.5%)
Positive	17 (20.5)

*Only 137 cases with available tumor type data **^#^**Available data detected.

### Immunohistochemistry

The TMA sections were immunostained according to a standard staining procedure described previously (Li et al., 2013). Mouse or rabbit IgG was used as a negative control. The percentage and intensity of staining was independently scored by two pathologists in a blinded manner. The staining intensity was scored for four categories: 0 (no visible staining), 1 (light brown staining), 2 (medium brown staining), and 3 (dark brown staining), with the same intensity covering more than 75% of the stained area. CD147 staining was scored as negative (no positivity at all); weak (1 + positivity regardless of positive cell percentages or 2 + positivity of ≤30% of cells); moderate (2 + positivity of >30% of cells or 3 + positivity of ≤50% of cells); and strong (3+, positivity of >50% of cells). pSTAT3 staining was classified as negative (no staining); weak (1 + staining in ≤50% of cells or 2 + staining in ≤25% of cells); moderate (1 + staining in >50% of cells, 2 + staining in 26–75% of cells or 3 + staining in ≤25% of cells); strong (2 + staining in >75% of cells or 3 + staining in >25% of cells) (Li et al., 2009, 2013). For data analysis, the immunostained TMA-sections were divided into two groups of low- *vs.* high-expression. The low-expression group included cases with intensity scores indicating negative and weak staining, whereas the high-expression group included cases with intensity scores indicating moderate and strong staining.

### Statistical Analysis

All data are expressed as mean ± standard deviation (SD) of three independent experiments. An independent Student’s *t*-test or one-way ANOVA was performed to compare the continuous variables between the two groups or more than two groups, respectively, while the categorical variables were analyzed using the χ^2^ test. The Kaplan–Meier method and the log-rank test were used to compare overall survival, defined as the time from a patient’s surgery until death, and recurrence survival, defined as the time of patients having recurrence until death. Patients who survived were censored at the time of their last follow-up. The Cox’s proportional hazards model was used to analyze the association of CD147 and/or pSTAT3 expression with patient survival/recurrence for the independent prognostic predictors. All statistical analyses were carried out with SPSS 22.0 (IBM) and Prism 6.0 software (GraphPad). ^∗^*P* < 0.05, ^∗∗^*P* < 0.01, and ^∗∗∗^*P* < 0.001 compared with the control group.

## Results

### Detachment Promotes CSC Features of Breast Cancer Cells

**FIGURE 1:**
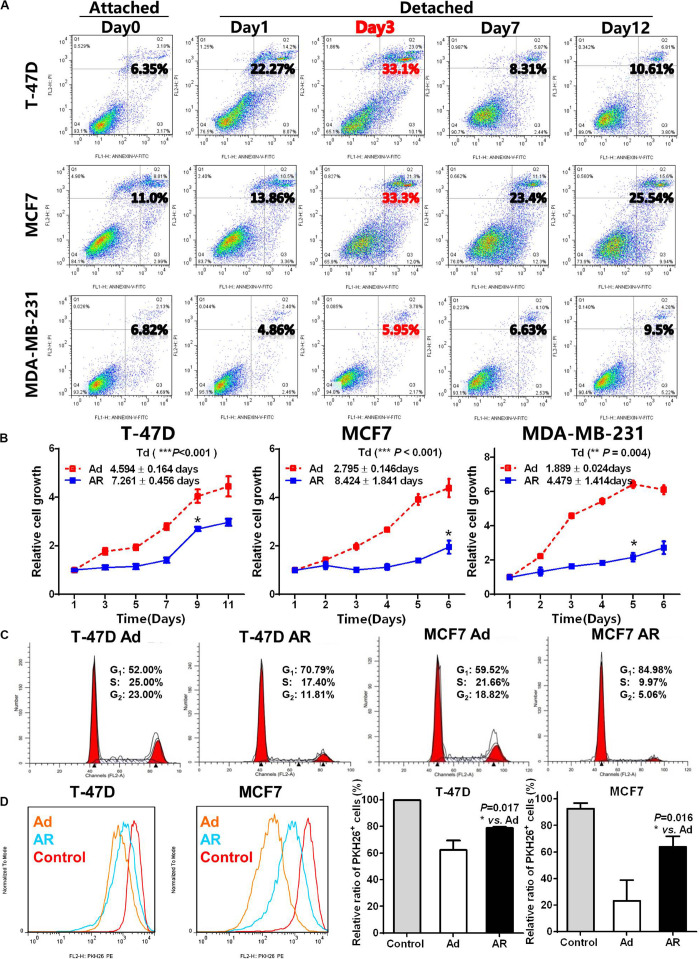
Detachment promotes CSC features of breast cancer cells. **(A)** Apoptosis analysis in breast cancer cells cultured under an attached condition in a common uncoated plate or a detached condition in ultra-low attachment plate for up to 12 days. **(B)** Cell growth measurement by detecting the WST-8 dye absorbance at 450 nm in breast cancer cells cultured under an adherent (Ad) or anoikis resistant (AR) condition for 7 days and then cultured under an adherent condition for up to 12 days. *T*_*d*_, doubling time. **(C)** Cell cycle distribution analysis in T-47D and MCF7 cells cultured under an Ad or AR condition for 7 days. **(D)** Cell proliferation analysis by PKH 26 staining in T-47D and MCF7 cells cultured under an Ad or AR condition for 7 days. Unlabeled cells were choosing as a control.

To detect the effect of detachment culture on induction of CSC potential, we first determined the ability of breast cancer cells to resist anoikis. As compared with cancer cells cultured under the attached condition, we found that cancer cells grown under the detached condition exhibited different degrees of apoptosis (Figure 1A). The detached MDA-MB-231 cells showed slight alterations in the apoptotic ratio compared with those of cells grown under the attached condition; however, both T-47D and MCF7 cells showed notable apoptosis on Day 3, which gradually reduced until Day 7, with 76–90% of cells surviving the detached condition. In the following experiments, we selected Day 3 as the cutoff time point for anoikis induction and Day 7 as the cut-off time point for cell survival (anoikis resistance).

Compared with the adherent (Ad) cells, anoikis resistant (AR) cells had a restoration of cell growth after 1–4 days of latent phase with varying degrees of prolonged doubling time (Td, Figure 1B). The reduced AR cell growth coincided with a higher proportion of cell in the G_1_-phase (Figure 1C and Supplementary Figure S1A), and a significantly higher number of PKH26 positive slow-cycling or quiescent cells compared to the Ad cells (Figure 1D). As anoikis resistance, slow-cycling, and quiescence are essential features of CSCs, we hypothesized that detachment may promote the acquisition of CSC potential in AR cells.

### Detachment Increases Functional ALDH^+^ or CD44^high^CD24^–/*low*^ CSCs

**FIGURE 2:**
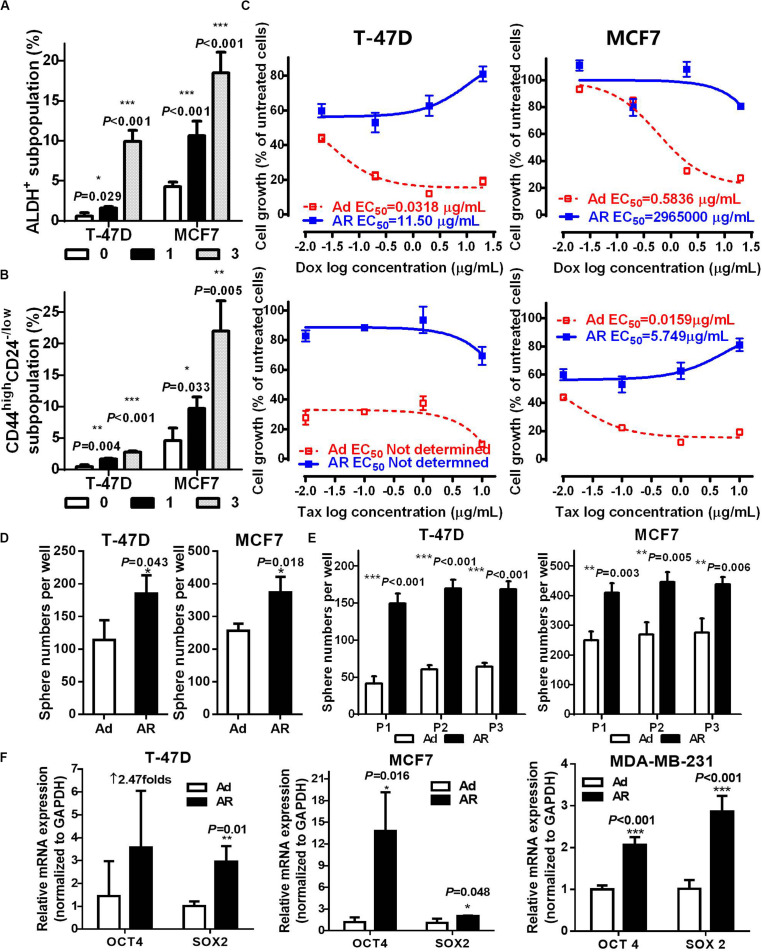
Detachment increases functional ALDH^+^ or CD44^high^CD24^– /*low*^ CSCs. ALDH^+^ **(A)** and CD44^high^CD24^– /*low*^ **(B)** subpopulation analysis in T-47D and MCF7 cells cultured under an attached or detached condition for 0, 1, or 3 days. **(C–F)** Cells were cultured under an Ad or AR condition for 7 days, and then used for the following experiments: **(C)** Cytotoxicity of Doxorubicin (Dox) and Taxol (Tax) in T-47D and MCF7 cells. **(D,E)** Tumorsphere numbers without or with passaging in T-47D and MCF7 cells. **(F)** qRT-PCR analysis of OCT4 and SOX2 mRNA levels in T-47D, MCF7, and MDA-MB-231 cells.

To assess whether AR cells acquire CSC potential upon detachment, we assayed the ratio of ALDH^+^ or CD44^high^CD24^–/*low*^ CSCs. As compared with that of attached cells, detached cells had a time-dependent increase in the ALDH^+^ subpopulation (Figure 2A and Supplementary Figure S1B). Under the detached condition, the ratio of CD44^high^CD24^–/*low*^ subpopulation increased in a time-dependent manner in the anoikis-sensitive T-47D and MCF7 cells but not in anoikis-insensitive MDA-MB-231 cells, which have a higher percentage of the CD44^high^CD24^–/*low*^ subpopulation (over 90%, Figure 2B and Supplementary Figure S1C). We further isolated fresh *ex vivo* malignant pleural effusion (MPE) cells from two breast cancer patients and cultured cells under attached or detached conditions for 14 days (Supplementary Figure S2A). Upon detachment, the ratio of the ALDH^+^ subpopulation increased by 1.91- and 2.12-fold in both MPE cells, while the ratio of the CD44^high^CD24^–/*low*^ subpopulation increased by threefold in the MPE cells (Supplementary Figures S2B,C). These data suggest that breast cancer cells cultured under the detached condition have increased CSCs.

Next, we determined the influence of detachment on CSC traits, including drug sensitivity, spherogenesis, and stemness. Compared to those of Ad cells treated with either Doxorubicin or Taxol, AR cells showed a significant increase of 50% effective concentration (EC_50_, more than 360-fold inductions; Figure 2C and Supplementary Figure S2D). AR cells from the T-47D and MCF7 were capable of growing in the medium containing Doxorubicin and Taxol, respectively. AR cells also formed significantly more tumor spheres compared to the Ad cells (1.66 ± 0.19 and 1.47 ± 0.30-fold increases in T-47D and MCF7 cells, respectively, Figure 2D), and had higher spherogenesis capacity that was maintained after the third passage of tumorsphere culture (Figure 2E), implying that detached culture enhanced the self-renewal potential of CSCs. Moreover, AR cells expressed a significantly higher level of stemness genes like OCT4 and SOX2 compared to the Ad cells (Figure 2F). These results demonstrate that detachment enhances functional CSCs in AR cells.

### Detachment Induces CSC Potential in ALDH^–^ or CD44^*low*^CD24^high^ Non-CSCs

**FIGURE 3:**
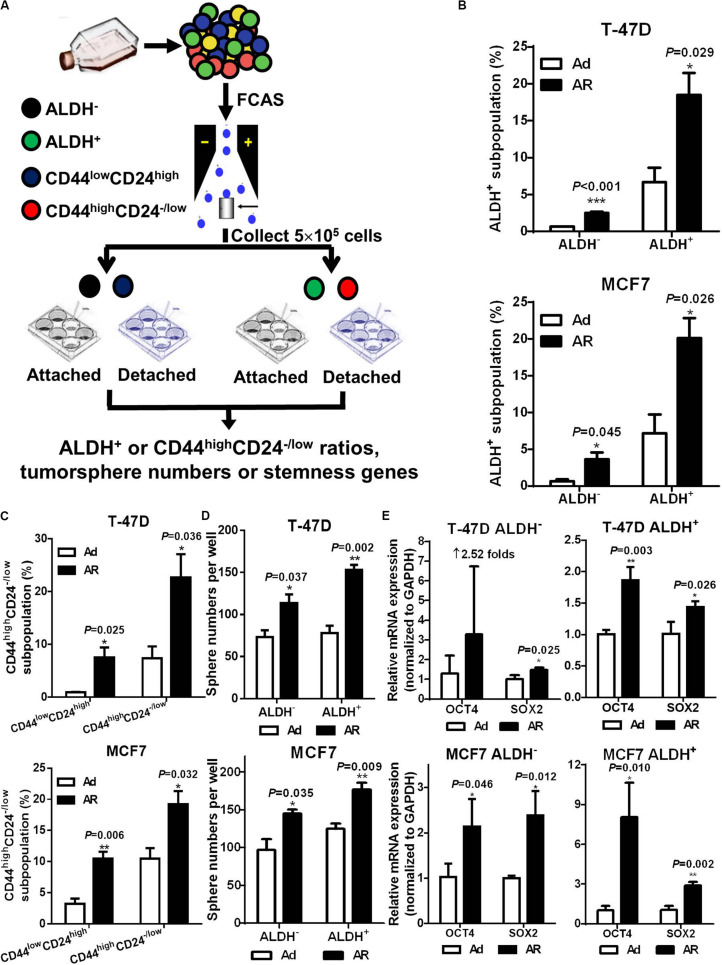
Detachment induces CSC potential in ALDH^–^ or CD44^*low*^CD24^high^ non-CSCs. **(A)** Study design. Non-CSCs (ALDH^–^ or CD44^*low*^CD24^high^) and CSCs (ALDH^+^ or CD44^high^CD24^– /*low*^) were sorted from T-47D and MCF7 cells and cultured under an Ad or AR condition for 7 days, then the ratio of ALDH^+^ or CD44^high^CD24^– /*low*^ subpopulation were analyzed. Flow cytometry analysis of ALDH^+^ **(B)** and CD44^high^CD24^– /*low*^ **(C)** subpopulation in non-CSCs and CSCs. Tumorsphere formation **(D)** and qRT-PCR analysis **(E)** of OCT4 and SOX2 mRNA levels in ALDH^–^ or ALDH^+^ subpopulation cultured under an Ad or AR condition for 7 days.

Previous studies revealed that differentiated cancer cells were able to acquire stem cell potential under environmental stress, such as hypoxia (Gammon et al., 2013), radiation (Peitzsch et al., 2016; Chen et al., 2017), and chemotherapy (Hu et al., 2012). In the following study, we explored whether non-stem breast cancer cells acquire CSC potential under a detached condition. We isolated non-CSCs (ALDH^–^ or CD44^*low*^CD24^high^) and CSCs (ALDH^+^ or CD44^high^CD24^–/*low*^), cultured them under either attached or detached conditions for 7 days, and then analyzed the CSC ratios (Figure 3A). Our data showed that upon detachment, both CSC and non-CSC subpopulations had significant increases in the ratio of CSCs (Figures 3B,C). However, the fold increases of CSC ratios were much higher in non-CSCs than in CSCs; the average increases of CSC ratios in CD44^*low*^CD24^high^ non-CSCs and in CD44^high^CD24^–/*low*^ CSCs obtained from T-47D cells were 7.90 ± 2.68 and 3.77 ± 2.68-fold, respectively; and 3.61 ± 1.57 and 1.90 ± 0.42-fold, respectively, for MCF-7 cells. These findings were similar to the results in the ALDH^–^ and ALDH^+^ subpopulations. Next, we isolated ALDH^–^ non-CSCs and ALDH^+^ CSCs, and cultured them under Ad or AR conditions for tumorsphere formation and stemness gene analysis. We found that tumorsphere formation ability and stemness gene expression, especially SOX2 mRNA levels, were significantly increased both in the ALDH^–^ non-CSCs and ALDH^+^ CSCs upon detachment, while OCT4 mRNA levels were significantly increased only in the ALDH^+^ CSCs upon detachment (Figures 3D,E). These data demonstrate that the CSC phenotypes and traits can be induced from non-CSCs under a detached condition.

### CD147 Mediates Detachment Induced CSC Potential in Non-CSCs

**FIGURE 4:**
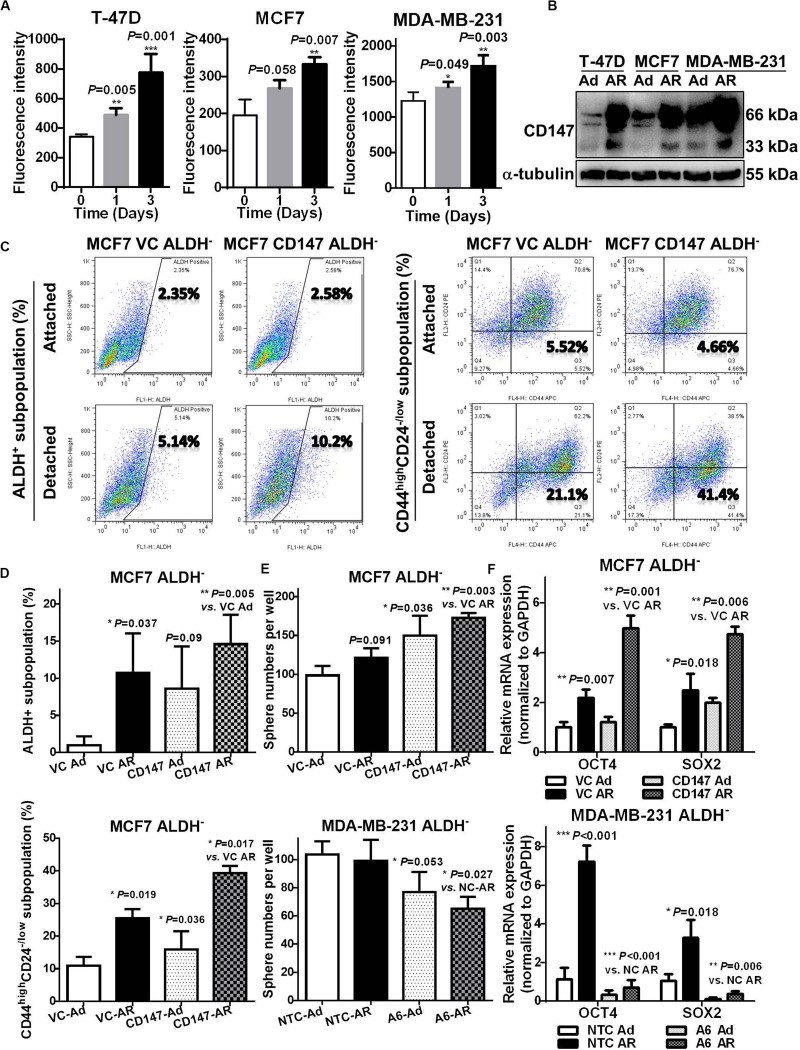
CD147 mediates detachment induced CSC potential in non-CSCs. **(A)** Flow cytometry analysis of membranous CD147 levels in T-47D, MCF7, and MDA-MB-231 cells cultured under an attached or detached condition for 0, 1, and 3 days. **(B)** Immunoblot assay of CD147 protein levels in T-47D, MCF7, and MDA-MB-231 cultured under an Ad or AR condition for 7 days. **(C,D)** ALDH^+^ and CD44^high^CD24^– /*low*^ subpopulation analysis in ALDH^–^ non-CSC subpopulation from CD147 knock-in (CD147) and control vector (VC) cells cultured under an Ad or AR condition for 7 days. Tumorsphere numbers **(E)** and OCT4 and SOX2 mRNA levels **(F)** in ALDH^–^ subpopulation with or without CD147 knock-in or knockdown (A6 and NTC) cultured under an Ad or AR condition for 7 days.

Our previous studies revealed that highly expressed CD147 in breast cancer tissues is an independent prognostic predictor for cancer recurrence and metastasis (Li et al., 2009), and CD147 induces the anoikis resistance of transformed HEK293 (Ma et al., 2010) and liver cancer cells (Ke et al., 2012). In our current breast cancer studies, we further confirmed that CD147 expression was associated with the ability to resist anoikis, with AR MDA-MB-231 cells having significantly higher CD147 expression compared to the anoikis sensitive T-47D and MCF7 cells (Supplementary Figures S2E,F, S3A *left*). Furthermore, upon detachment, CD147 protein levels were upregulated in a time-dependent manner in all three cell lines, although CD147 mRNA levels only increased in the T-47D and MCF7 cells (Figures 4A,B and Supplementary Figure S3A *right*).

To explore the influence of CD147 on detachment-induced CSC potential, we constructed the CD147 knockdown (A6)/negative control (NTC) or CD147 overexpressing (CD147)/vector control (VC) breast cancer cells and cultured them in a detached condition. As shown in Supplementary Figures S3B–G, effectively silencing CD147 expression (>70%) resulted in sensitizing MDA-MB-231 cells to detachment-induced apoptosis, while CD147 overexpression greatly enhanced the MCF7 cells’ ability to resist anoikis. CD147 knockdown significantly decreased tumorsphere numbers both in a passage-dependent manner and at a single cell level (Supplementary Figure S4A). Compared to NTC cells, CD147 knockdown A6 cells had a much lower tumor incidence (2/8 *vs*. 5/8), an increased median tumor formation time (>44 *vs*. 25 days), and significantly smaller tumors (77.05–81.72% reduction between Day 20 and Day 44, Supplementary Figure S4B). CD147 knockdown also abolished the detachment-induced ALDH^+^ subpopulation (0.43-fold *vs.* 1.63-fold), while CD147 overexpression promoted the detachment-induced stemness by upregulating SOX2 expression (Supplementary Figures S4C,D).

Next, we examined whether detachment-induced CD147 expression could help generate CSCs in non-CSCs. As shown in Figures 4C,D, CD147 knock-in significantly boosted detachment in both ALDH^+^ and CD44^high^CD24^–/*low*^ subpopulation in MCF7 ALDH^–^ cells. Moreover, CD147 overexpression significantly increased whereas CD147 knockdown remarkably abolished the detachment-induced spherogenesis and stemness in ALDH^–^ MCF7 and MDA-MB-231 cells (Figures 4E,F). These data suggest that CD147 is associated with CSC traits in AR breast cancer cells, and that it plays an essential role in detachment-induced conversion of non-CSCs to CSCs.

### CyPA Induces CSC Potential in Non-CSCs via Activating CD147

**FIGURE 5:**
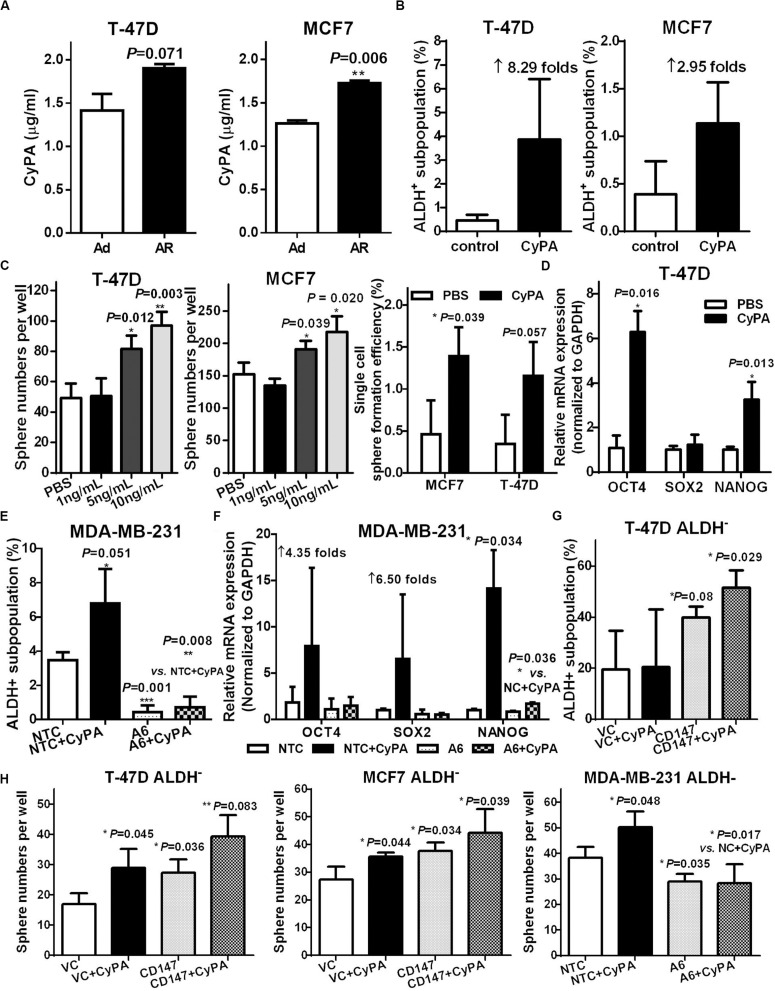
CyPA induces CSC potential in non-CSCs *via* activating CD147. **(A)** Supernatant CyPA levels from T-47D and MCF7 cells cultured under an attached or detached condition for 3 days. ALDH^+^ subpopulation analysis **(B)** and tumorsphere formation **(C)** in T-47D and MCF7 cells cultured with or without CyPA. **(D)** OCT4, SOX2, and NANOG mRNA level detection in T-47D cells cultured with or without CyPA. The ratio of ALDH^+^ subpopulation **(E)** and OCT4, SOX2, and NANOG mRNA level **(F)** analysis in MDA-MB-231 A6 and NTC cells cultured with or without CyPA. **(G)** ALDH^+^ subpopulation analysis in ALDH^–^ subpopulation from T47D CD147 and VC cells cultured with or without CyPA. **(H)** Tumorsphere number analysis in ALDH^–^ subpopulation with or without CD147 knock-in or knockdown cultured with or without CyPA. Apart from the dose-dependent tumorsphere formation assay, all cells used for the above experiments were treated with 20 ng/mL CyPA for 24 h.

Cyclophilin A (CyPA) is a natural CD147 ligand and functions as an autocrine/paracrine chaperone molecule to facilitate CD147 membrane expression and stabilization for CD147 activation in cells (Zhu et al., 2015). Therefore, we explored whether CyPA induced CSC potential in breast cancer cells that were cultured under a detached condition. We found that the conditioned medium of AR cells had more extracellular CyPA (eCyPA) compared to the conditioned medium of the Ad cells (Figure 5A and Supplementary Figure S4E). We further found that recombinant CyPA dose-dependently increased the CSC subpopulation, tumorsphere formation, and stemness gene expression (Figures 5B–D and Supplementary Figure S4F). Together, these results suggest that eCyPA induces CSC potential.

We next investigated whether the CyPA-induced CSC potential was mediated by CD147 activation through altering the level or activity of CD147 or CyPA. Our data showed that knockdown of CD147 significantly restrained the CyPA induced ALDH^+^/CD44^+^CD24^–/*low*^ subpopulation and stemness gene expression (Figures 5E,F and Supplementary Figures S5A,B). Conversely, CyPA inhibition reduced the CD147-overexpression increased ALDH^+^ and CD44^+^CD24^–/*low*^ subpopulations (Supplementary Figures S5C–F). More importantly, CD147 overexpression further increased, whereas CD147 knockdown significantly blocked the CyPA-induced ALDH^+^ subpopulations and spherogenesis in ALDH^–^ non-CSCs (Figures 5G,H). These data support that CyPA-activated CD147 mediates detachment-induced CSC potential in non-stem breast cancer cells.

### CD147-STAT3 Signaling Contributes to Induced CSC Potential and Predicts Poor Prognosis and Recurrence in Breast Cancer Patients

**FIGURE 6:**
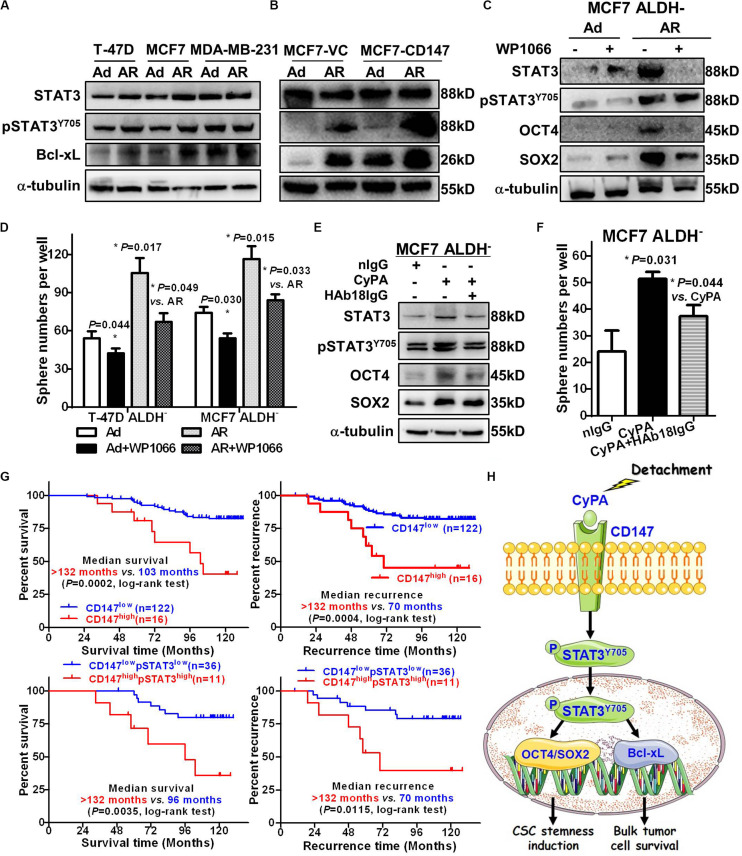
CD147-STAT3 signaling contributes to induced CSC potential and predicts poor prognosis and recurrence in breast cancer patients. STAT3, pSTAT3^Y705^, and Bcl-xL protein levels in T-47D, MCF7, and MDA-MB-231 cells **(A)** and MCF7-CD147/VC cells **(B)** cultured under an Ad or AR condition for 7 days. α-tubulin was used as a loading control and one representative of three biological replicates is shown. STAT3, pSTAT3^Y705^, OCT4, and SOX2 protein levels **(C)** and tumorsphere numbers **(D)** in ALDH^–^ cells treated with or without 1 μM WP1066 for 24 h and then cultured under an Ad or AR condition for 7 days. STAT3, pSTAT3^Y705^, OCT4, and SOX2 protein levels **(E)** and tumorsphere numbers **(F)** in ALDH^–^ cells treated with or without 20 ng/mL CyPA and 10 μg/mL HAb18IgG for 24 h. **(G)** Kaplan–Meier curve analysis of overall survival and tumor recurrence time in breast cancer patients stratified by level of cytoplasmic CD147 and/or pSTAT3. *Up:* Overall survival (left) and tumor recurrence time (right) in 138 patients stratified by low- *vs.* high CD147 expression. *Down:* Overall survival (left) and tumor recurrence time (right) in 47 patients stratified by low- *vs.* high CD147/pSTAT3 expression. **(H)** A proposed working model for detachment-induces CSC potential in non-stem breast cancer cells *via* CyPA-CD147-pSTAT3 signaling.

We previously reported that CD147 induces cell proliferation and invasion by activating STAT3 signaling (Li et al., 2013; Xu et al., 2016), while others showed that STAT3 is associated with anoikis resistance (Fofaria and Srivastava, 2015) and the conversion of non-CSCs to CSCs (Kim et al., 2013). Thus, we next determined the role of STAT3 signaling in CD147 mediated conversion of non-CSCs to CSCs upon detachment. We observed that, compared to anoikis-sensitive T-47D and MCF7 Ad cells, AR cells had upregulated protein levels of total and phosphorylated STAT3 as well as its downstream target Bcl-xL (Figure 6A). We also found that the detachment-increased STAT3 signaling occurred in a CD147-dependent manner, as CD147 overexpression or knockdown dramatically enhanced or reduced the detachment-induced pSTAT3/Bcl-xL level, respectively (Figure 6B and Supplementary Figure S6A). Furthermore, the STAT3 inhibitor WP1066 significantly attenuated the detachment-induced spherogenesis and stemness by downregulating SOX2 protein expression in the detached ALDH^–^ non-CSCs (Figures 6C,D). We also found that HAb18IgG, a CD147 antibody, significantly reversed CyPA-induced STAT3 signaling activation, downstream SOX2/OCT4 stemness gene expression, and sphere formation in ALDH^–^ non-CSCs, which demonstrates that targeting CyPA-CD147 signaling could suppress STAT3 signaling and inhibit the generation of CSCs (Figures 6E,F). Taken together, our data indicate that CD147 promotes detachment-induced conversion of non-CSCs to CSCs by activating STAT3 signaling.

To investigate whether CD147-pSTAT3 signaling has a clinicopathological connection, we correlated patient survival and recurrence data with CD147 and pSTAT3 levels in breast cancer tissues from 138 patients. The patients with high cytoplasmic but not membranous CD147 expression had poorer overall survival and tumor recurrence (Figure 6G and Supplementary Figure S6B). The double immunofluorescence staining showed that CD147 and pSTAT3^Y705^ were co-expressed in breast cancer tissues (Supplementary Figure S6C). Furthermore, the co-expression of CD147 and pSTAT3^Y705^ was higher in detached cells than in adherent cells (Supplementary Figure S6D). These data are consistent with our recent reports that CD147 undergoes endocytosis, which removes membrane proteins in the cytoplasm, releases the intracellular domains in the nucleus, and contributes to liver cancer progression and poor prognosis (Wu et al., 2017; Yong et al., 2019). Moreover, cytoplasmic CD147 expression was significantly associated with pSTAT3 expression (Spearman *r* = 0.184, *P* = 0.03; Supplementary Figure S6E). Patients with high cytoplasmic CD147 and pSTAT3 co-expressed tumors had worse overall survival and tumor recurrence (Figure 6G and Table 2). The multi-variant Cox proportional hazards analysis shows that co-expression of cytoplasmic CD147 and pSTAT3 was an independent predictor for poor overall survival after adjusting for tumor type/grade, pT/pN/pM/stage, and clinical stages (hazard ratio = 31.85; 95% confidence interval, 2.67–380.02; *P* = 0.006, Table 3).

**TABLE 2:** Association of CD147/pSTAT3 co-expression with clinicopathological features.

Clinicopathological features	CD147^*low*^ pSTAT3^*low*^^∗^ (*n* = 36)	CD147^high^ pSTAT3^high^^∗^ (*n* = 11)	*P* value^†^
Age (*n*, ≤ 50/ > 50 years old)	16/20	5/6	0.95
Tumor type (*n*, IDC/others)	32/4	10/1	1
Tumor grade (*n*, II/II-III + III)	26/10	5/6	0.20
pT stage (*n*, T1/T2 + T3)	8/28	4/7	0.58
pN stage (*n*, N0/N1 + N2 + N3)	18/18	5/6	1
pM stage (*n*, M0/M1)	33/3	11/0	0.77
AJCC stage (*n*, 1/2A + 2B + 3A + 3C)	5/31	0/11	0.45
Patient survival (*n*, alive/dead)	29/7	5/6	***0.023***
Recurrence (*n*, no/yes)	11/25	4/7	1

*Low (negative to weak expression), high (moderate to strong expression). ^†^Estimated by χ^2^ test. Bold italic values specified in P values are statistically significant.

**TABLE 3:** The Cox proportional hazard analysis of independent predicators for overall survival of breast cancer patients.

Clinicopathological features	HR (95% CI)	*P* value^∗^
Cytoplasmic CD147/pSTAT3 co-expression (CD147^*low*^pSTAT3^*low*^, CD147^high^pSTAT3^high^)	31.85 (2.67–380.02)	***0.006***
Age (≤50, >50 years old)	9.35 (1.22–71.64)	***0.031***
Tumor type (IDC, ILC + mucinous adeno + others)	4.46 (0.32–61.18)	0.26
Tumor grade (II, II–III + III)	1.74 (0.32–9.40)	0.51
pT stage (T1, T2 + T3)	19.92 (0.80–491.57)	0.067
pN stage (N0, N1 + N2 + N3)	1.80 (0.24–13.35)	0.56
pM stage (M0, M1)	0.73 (0.036–14.63)	0.83
AJCC Stage (1, 2A + 2B + 3A + 3C)	0.73 (0.036–14.63)	0.99
Recurrence (no, yes)	11.97 (1.59–90.07)	***0.016***

HR, hazard ratio; CI, confidence interval. *P < 0.05. Bold italic values specified in P values are statistically significant.

## Discussion

In the current study, we investigated the generation of induced breast cancer CSCs and the underlying molecular mechanisms under detachment culture conditions. We found that the resistance to detachment induced anoikis was associated with CSC characteristics, and detached culture was able to increase functional ALDH^+^ or CD44^high^CD24^–/*low*^ CSC subpopulations and to induce CSC potential in ALDH^–^ or CD44^*low*^CD24^high^ non-CSCs. Our data further demonstrated that the expression of CD147 and the secretion of its ligand CyPA were both enhanced in the detachment cultured breast cancer cells or supernatants, respectively, and high levels of CyPA/CD147 mediated the conversion of non-CSCs into CSCs *via* activating pSTAT3 signaling. In breast cancer tissue specimens, cytoplasmic CD147 and pSTAT3 were both highly expressed, and their concurrent expression was correlated with patient poor overall survival and tumor recurrence. In conclusion, our study provides exciting novel evidence that cancer cells cultured in a detached condition acquire stemness traits *via* upregulating CD147 expression. This suggests that detachment cultured non-stem cancer cells resistant to anoikis could be a source of induced CSCs with metastatic potential or MICs, and also indicates that targeting CD147 could be a promising therapeutic strategy for lethal metastatic breast cancer by eliminating induced CSCs.

In the metastasis cascade, the spread of a detached primary tumor cells through the bloodstream as CTCs is a critical step, and only CTCs enriched with stem-like features are considered the precursors of metastasis lesions. Therefore, the majority of studies have focused on the contribution of stem-like properties in CTCs to the metastatic initiating capacity (Gkountela and Aceto, 2016; Cortes-Hernandez et al., 2019). For example, the study by Gkountela et al. displayed the dedication of epigenetic regulation of stemness in detached CTC clusters on their metastasis-prone properties (Cortes-Hernandez et al., 2019; Gkountela et al., 2019). However, it is still not well known how CTCs acquire stemness potential. Using patient MPE derived cell lines and MPE cells grown in aggregates under detached conditions as a “surrogate” model for CTC clusters (May et al., 2018), we revealed that the CSC potential could be induced in breast cancer cells under a detached condition. Our current data indicate that detachment acts as a harsh microenvironmental pressure to induce CTC stemness potential. Therefore, the detailed signaling and molecular profiling involved in detachment induced stem-like potential holds exceptional promise to develop metastasis-tailored therapies.

Recently, the concept of phenotypic plasticity in cancer cells has been well established. Several studies confirmed that both CSCs and non-CSCs were capable of undergoing phenotypic transitions in response to microenvironmental stresses, with a dynamic equilibrium of dedifferentiation-redifferentiation between non-CSCs and induced CSCs (Batlle and Clevers, 2017; O’Brien-Ball and Biddle, 2017; May et al., 2018; Clarke, 2019). Specifically, non-CSCs can be induced or dedifferentiate into induced CSCs by radio-chemotherapy (Peitzsch et al., 2016; Chen et al., 2017) and also by detachment condition, as shown in this paper. As we prepared this manuscript for publication, Fumagalli et al. (2020) reported that Lgr5^–^ non-CSCs are able to convert into Lgr5^+^ CSCs at the metastatic site for efficient colorectal cancer metastatic outgrowth. Based on these finding, it seems that a successful cancer cure can be achieved only by killing both preexisting CSCs and induced CSCs. The key point to eradicate the induced CSCs is to prevent the formation of induced CSCs and to block the signaling pathways involved in de-differentiation of non-CSCs to CSCs. Thus, blocking the detachment of cancer cells from the ECM and the subsequent induction of CSC potential could be a novel strategy for therapeutic intervention of cancer metastasis.

Due to their plasticity, CSCs will always be activated or recreated on the condition that the CSC niche remains intact. Thus, a better understanding of the underlying genetic and epigenetic factors in the niche where cancer cells adopt phenotypic plasticity could help us to effectively control cancer progression and recurrence (O’Brien-Ball and Biddle, 2017). For example, a cancer pre-metastatic niche, like the MPE, may function as a dynamic reservoir for invasive cancer cells, where cancer cells may establish a malignant cytokine network for their survival and metastasis (Giarnieri et al., 2015; Bruschini et al., 2020). In our current study, we revealed that the levels of eCyPA in the conditioned medium of detachment cultured cells were significantly higher than that in cells growing under the attached condition; while the addition of recombinant CyPA promoted the conversion of cancer cells to CSCs in a CD147-dependent manner. Previous studies showed that IL-6 can induce conversion of non-CSCs to CSCs (Iliopoulos et al., 2011; Kim et al., 2013), but we did not detect any increases in IL-6 levels in our experimental conditions (data not shown). Although cytoplasmic CyPA is reported to play a role in maintaining glioma CSC stemness (Wang et al., 2017), our study is the first report of such effort to focus on the effects of extracellular secreted CyPA on induction of CSC potential. Unfortunately, it remains unknown how and why non-CSCs were able to produce eCyPA and overexpress CD147 upon detachment. Future studies are needed to identify the upstream signaling cascade that triggers the expression and activation of eCyPA and CD147 in breast cancer cells growing under the detached condition.

Previous studies revealed that high CD147 expression on cancer cells is linked to CSC characteristics, such as EMT, anoikis-resistance, and chemoresistance (Ma et al., 2010; Wu et al., 2011; Ke et al., 2012; Dai et al., 2013), indicating that cancer cells expression high levels of CD147 could be more aggressive and prone to cancer metastasis and recurrence. Indeed, our previous findings indicated that anti-CD147 HAb18IgG sensitized pancreatic cancer cells to chemo-radiotherapy and reduced CSC potential by inhibiting pSTAT3 signaling (Fan et al., 2019), and the CD147 antibody drug, namely, Licartin, was successfully used to prevent tumor recurrence after liver transplantation or radiofrequency ablation in advanced hepatocellular carcinoma patients (Xu et al., 2007a; Bian et al., 2014). Here, we demonstrate that CD147 expression on breast cancer cells was upregulated when culturing cells under a detached condition, and CD147 expression boosted the detachment-induced conversion of non-CSCs to CSCs by activating STAT3-SOX2 signaling. In this regard, blocking circulating breast cancer cells with an anti-CD147 antibody could be clinically effective at suppressing the induced CSCs (or MICs), and subsequently preventing the generation of metastatic tumor mass.

## Conclusion

In summary, our current study demonstrates that detachment induced breast cancer cells to express CD147 and secrete CyPA, which played an important role in the conversion of non-CSCs to CSCs through activation of the STAT3-SOX2 signaling pathway (Figure 6H). Thus, our findings uncovered a pivotal role of the eCyPA-CD147 axis in the functional link between anoikis resistance and induced CSC potential. The translational extension of these studies into the clinical setting could include the development of novel therapies of breast cancer metastasis by targeting the eCyPA–CD147 interaction.

## Data Availability Statement

All datasets presented in this study are included in the article/Supplementary Material.

## Ethics Statement

The animal study was reviewed and approved by the Animal Care and Use Committee of The Fourth Military Medical University.

## Author Contributions

LL, J-LJ, and Z-NC conceived the study and participated in the study design, performance, coordination, and manuscript writing. YM, X-YF, L-JY, B-QX, DH, H-YC, S-JW, and L-JW obtained the samples, performed the experiments, interpreted the data, and prepared the figures. X-QW and LX revised the manuscript. All authors reviewed and approved the final manuscript.

## Conflict of Interest

The authors declare that the research was conducted in the absence of any commercial or financial relationships that could be construed as a potential conflict of interest. The reviewer YL declared a shared affiliation with the authors to the handling editor at the time of review.
