# Supplementary material for: Detachment Activated CyPA/CD147 Induces Cancer Stem Cell Potential in Non-stem Breast Cancer Cells
Source: Front Cell Dev Biol. 2020 Oct 16;8:543856. doi: 10.3389/fcell.2020.543856 (PMC7640948; doi:10.3389/fcell.2020.543856)
Supplement: Supplementary file 1 [file Data_Sheet_1.doc]

**Supplementary Materials**

**Materials and Methods**

***Antibodies and reagents***

Antibodies against SOX2, phospho-STAT3 (Tyr^705^) and total STAT3 were obtained from Cell Signaling Technology (Danvers, MA), OCT4 and Bcl-xL from Abcam (Cambridge, MA), Mcl-1 from Proteintech (Rosemont, IL), α-tubulin from Santa Cruz Biotechnologies (Santa Cruz, CA). APC-conjugated anti-human CD44, PE-conjugated anti-human CD24, PerCP-conjugated anti-human CD147, and isotype-matched mouse immunoglobulin were from BD Biosciences (San Jose, CA). Goat anti-rabbit IgG conjugated with horseradish peroxidase (HRP), goat anti-mouse IgG conjugated with HRP, and Lipofectamine 2000 transfection reagent were from Invitrogen (Carlsbad, CA). Puromycin was from InvivoGen (San Diego, CA), while recombinant human CyPA was from Sigma Chemicals (St. Louis, MO) and CyPA inhibitor TMN 355 was from Tocris (Minneapolis, MN). Doxorubicin and Taxol were purchased from Lilly France S.A. (Fegersheim, France) and WP1066 were purchased from Calbiochem (Billerica, MA). Anti-mouse CD147 antibody HAb18 IgG was prepared as previously reported .

***Processing of malignant pleural effusion (MPE)***

The MPE samples were obtained from the pleural cavity of breast cancer patients and MPE cells were isolated as reported by DeRose et al and then analyzed using cytology for confirmation of breast cancer diagnosis. This study was approved by the Institutional Review Board of The Air Force Medical University. In brief, the pleural effusion was centrifuged at 530 g for 5 min at 4^o^C and cell pellets were suspended in 5 to 10 ml of Red Cell Lysis Buffer (Tiangen Biotech, Beijing, China) to remove erythrocytes, and the layer of mononuclear cells was then collected and washed twice with 3 volumes of Hank's Balanced Salt Solution (HBSS) through centrifugation at 400 g for 10 min at the room temperature. Next, cell pellets were resuspended in 2% FBS/HBSS with 1 μg/ml 7-AAD (BD Biosciences) to gate the viable cells for further experiments.

***Cell growth assay***

Cells were seeded at a density of 1-3 × 10^3^ cells per well in five replicates into 96-well common plates, and incubated for indicated times. Cell growth was assessed using the Cell Counting Kit-8 containing WST-8 (Beyotime Biotechnology, Beijing, China) according to a previous study . The optical density values were measured with a microplate reader at 450 nm. The cell doubling time (amount of doubling in one unit of time) was calculated by the cell growth rate (*μ*) using the equation t_d_=, as described previously (Li et al., 2011). In the equation, *μ* =$\frac{LnX_{2}-LnX_{1}}{t_{2}-t_{1}}$, *X* was fold change of A_450nm_ (calculated in relation to A_450nm_ on Day 1), *t* was culture time.

***Cell cycle assay***

For cell cycle distribution assay, 1 × 10^6^ cells were trypsinized and fixed with 70% cold ethanol at 4°C overnight. On the next day, fixed cells were re-suspended in 200 μl of PBS containing 0.5 mg/ml RNase, 0.05% Triton X-100 and 10 μg/ml PI, incubated at 37°C in the dark for 1 hour, and analyzed using a BD FACS Calibur Flow Cytometer.

***PKH26 staining***

For PKH26 staining, 5 × 10^5^ freshly harvested cells were washed with 10% FBS containing RPMI 1640 medium and incubated in suspension with 4 μM PKH26 (Sigma Chemicals) in 25 μl of Diluent C at the room temperature for 4 min. The excess dye was scavenged by addition of equal volume FBS and incubated for 1 min, followed by three washes in 10 ml RPMI-1640 containing 10% FBS through centrifugation. Thereafter, the cells were further cultured for 7 days for propagation and mitotic dilution of dye and then were harvested and analyzed by using BD FACS Calibur Flow Cytometer.

***Drug toxicity assay***

The chemo-sensitivity was assayed by using EC_50_ values. In brief, 3 × 10^3^ cells per well were seeded into 96-well plates and grown overnight, and treated with 0.02-20 μg/mL Doxorubicin or 0.01 - 10 μg/mL Taxol for 72 h, and then subjected to the MTT assay and calculation of the EC_50_ values using GraphPad Prism 6.0 (GraphPad Software, La Jolla, CA). Herein, the cells incubated with absolute alcohol (solvent for Taxol) and PBS (solvent for Doxorubicin) were considered as untreated cells. Untreated cells cultivated for 3 days were chosen as a control.

***Quantitative reverse transcriptase-polymerase chain reaction (qRT-PCR)***

Total cellular RNA was isolated using Trizol and reversely transcribed into cDNA, and qPCR then was ampliﬁed by using the Stratagene Mx3005P Multiplex quantitative PCR system (Agilent Technologies, Santa Clara, CA) with primers according to a previous study . The primer sequences were CD147, 5'-ACTCCTCACCTGCTCCTTGA-3' and 5'-GCCTCCATGTTCAGGTTCTC-3'; OCT4, 5'-GGGCTCTCCCATGCATTCAAAC-3' and 5'-ACCTTCCCTCCAACCAGTTGC-3'; SOX2, 5'-TGGACAGTTACGCGCACAT-3' and 5'-CGAGTAGGACATGCTGTAGGT-3'; NANOG, 5'-CCCCAGCCTTTACTCTTCCTA-3' and 5'-CCAGGTTGAATTGTTCCAGGTC-3'; and GAPDH, 5'-TGATGACATCAAGAAGGTGGTGAAG-3' and 5'-TCCTTGGAGGCCATGTGGGCCAT-3'. The relative mRNA levels were calculated as unit values of 2^−ΔCt^ = 2^−(Ct (HKG) − Ct (GOI))^.

***Immunoblotting***

Whole cell lysis was prepared using the phospho-RIPA buffer (1 M Tris-HCl at pH 7.5, 5 M NaCl, 0.01% NP-40, 0.5 M EGTA, and 10% SDS) supplemented with a complete EDTA-free protease inhibitor cocktail (Roche, Indianapolis, IN). After quantitation, protein samples were separated in sodium dodecyl sulfate-polyacrylamide gel electrophoresis (SDS-PAGE) gels and transferred onto polyvinylidene fluoride membranes (PVDF; Millipore). For immunoblotting, the membranes were blocked in 5% BSA/PBS solution at the room temperature for 1 h and then were incubated with each individual primary antibody and subsequently, with horseradish peroxidase-conjugated anti-mouse or anti-rabbit IgG. After that, protein signals were developed using the Enlight Western blot ECL reagents (Engreen, Beijing, China) and level of α-tubulin was used as a loading control.

***Flow cytometry analysis of CD147 membrane expression***

To detect level of membranous CD147 expression, 1 × 10^6^ cells were incubated with a PerCP-conjugated anti-human CD147 antibody in HBSS containing 2% FBS at 4°C for 30 min, while the isotype-matched PerCP mouse IgG was used as a negative control. After washed with HBSS thrice, the cell samples were analyzed using FACS Calibur Flow Cytometer.

***Enzyme-linked immunosorbent assay (ELISA)***

To measure the level of secreted CyPA, the culture supernatants were collected and the cell number in each well was counted. Levels of CyPA were detected by using a CyPA ELISA Kit (Shanghai Enzyme-linked Biotechnology Co., Shanghai, China) according to the manufacturers’ protocols and the data were normalized as 1 × 10^5^ cells in each sample according to the Standards from the kits.

***Colony formation assay***

For colony formation assay, 100, 200, 500 cells per well were cultured with RPMI-1640 medium containing 10% FBS in 6-well plates for 7 - 10 days. Then, the colonies were stained by 0.1% crystal violet and counted (> 50 cells) manually under an inverted microscope.

***Animal studies***

Animal experiments were approved by the Institutional Animal Care and Use Committee of The Air Force Medical University. Briefly, five- to six-week-old female athymic NCr-nu/nu nude mice were obtained from Silaikejingda Experimental Animal Co., Ltd (Hunan, China) and subcutaneously inoculated on both ﬂanks with 1 × 10^6^ MDA-MB-231 NTC or CD147 knock-down A6 cells in 0.2 mL DMEM (n = 10 per group). Mice were monitored for tumor formation for 6 weeks and tumor cell xenograft volume was measured regularly according to a previous study to calculate and record data .

***Immunofluorescence staining***

For tissue and cellular immunofluorescence staining, slides were incubated with primary antibody against CD147 and pSTAT3^Y705^ at 4°C overnight, and then followed by the incubation of Alexa Flour 555-conjugated donkey anti-mouse IgG and Alexa Flour 488-conjugated donkey anti-rabbit IgG at room temperature for 30 minutes. Sections were counterstained with DAPI in mounting medium. Images were captured with a Nikon microscope and were analyzed using Nikon NIS-Elements Advanced Research software.

**References**

DeRose, Y.S., Gligorich, K.M., Wang, G., Georgelas, A., Bowman, P., Courdy, S.J., et al. (2013). Patient-derived models of human breast cancer: protocols for in vitro and in vivo applications in tumor biology and translational medicine. *Curr Protoc Pharmacol* Chapter 14**,** Unit14 23. doi: 10.1002/0471141755.ph1423s60.

Li, L., Qin, J., Feng, Q., Tang, H., Liu, R., Xu, L., et al. (2011). Heparin promotes suspension adaptation process of CHO-TS28 cells by eliminating cell aggregation. *Mol Biotechnol* 47(1)**,** 9-17. doi: 10.1007/s12033-010-9306-1.

Li, L., Tang, W., Wu, X., Karnak, D., Meng, X., Thompson, R., et al. (2013). HAb18G/CD147 promotes pSTAT3-mediated pancreatic cancer development via CD44s. *Clin Cancer Res* 19(24)**,** 6703-6715. doi: 10.1158/1078-0432.CCR-13-0621.

Xu, B.Q., Fu, Z.G., Meng, Y., Wu, X.Q., Wu, B., Xu, L., et al. (2016). Gemcitabine enhances cell invasion via activating HAb18G/CD147-EGFR-pSTAT3 signaling. *Oncotarget* 7(38)**,** 62177-62193. doi: 10.18632/oncotarget.11405.

Xu, J., Shen, Z.Y., Chen, X.G., Zhang, Q., Bian, H.J., Zhu, P., et al. (2007). A randomized controlled trial of Licartin for preventing hepatoma recurrence after liver transplantation. *Hepatology* 45(2)**,** 269-276. doi: 10.1002/hep.21465.
