# Supplementary material for: Detachment Activated CyPA/CD147 Induces Cancer Stem Cell Potential in Non-stem Breast Cancer Cells
Source: Front Cell Dev Biol. 2020 Oct 16;8:543856. doi: 10.3389/fcell.2020.543856 (PMC7640948; doi:10.3389/fcell.2020.543856)
Supplement: Supplementary file 2 [file Data_Sheet_2.doc]

**Supplementary figure legends**

**Figure S1. Detachment increases ALDH^+^ or CD44^high^CD24^-/low^ CSCs. (A)** Cell cycle distribution analysis in CD147 knock-down MDA-MB-231 A6 and non-target control (NTC) cells cultured under an adherent (Ad) or detachment (AR) condition for seven days. **(B-C)** Flow cytometry analysis of ALDH^+^ **(**B**)** or CD44^high^CD24^-/low^ **(**C**)** subpopulation in MDA-MB-231 cells cultured under an attached or detached condition for 0, 1 or 3 days. For graphs, error bars represent the standard deviation of three technical replicates of three biological replicates. **P*< 0.05, ** *P*< 0.01, *** *P*< 0.001.

**Figure S2. Detachment increases functional ALDH^+^ or CD44^high^CD24^-/low^ CSCs. (A)** MPE-fractionation strategy. The MPE derived cells were cultured under an Ad or AR condition for 14 days and then subjected to flow cytometry analysis of the ratio of ALDH^+^ or CD44^high^CD24^-/low^ subpopulations. **(B-C)** ALDH^+^ (B) and CD44^high^CD24^-/low^ (C) subpopulations analysis in fresh isolated MPE cells cultured under an Ad or AR condition. (**D)** Apoptosis analysis by Annexin V/PI assay in MCF7 cells cultured under an Ad or AR condition for seven days and then treated with or without Doxorubicin (2 μg/mL) or Taxol (1 μg/mL) for two days. **(E-F)** Apoptosis analysis by Annexin V/PI assay (E) and membranous CD147 levels by flow cytometry analysis (F) in T-47D and MCF7 cells as compared to MDA-MB-231 cells, which cultured under detached condition for three days.

**Figure S3. CD147 is associated with anoikis resistance. (A)** Immunoblot assay and RT-PCR analysis of CD147 protein and mRNA levels in T-47D, MCF7 and MDA-MB-231 cells cultured under an attached or detached condition for three days. **(B)** Immunoblot assay of CD147 protein levels in MDA-MB-231 A6 and NTC cells. **(C)** Flow cytometry analysis of membranous CD147 levels in MDA-MB-231 A6 and NTC cells. Parent MDA-MB-231 and CD147 knock-out liver cancer cells K7721 were used as positive and negative control, respectively. **(D)** Immunoblot assay of CD147 protein levels in T-47D and MCF7 CD147 overexpressed (CD147) or vector control (VC) cells. **(E)** Flow cytometry analysis of membranous CD147 levels in T-47D and MCF7 CD147 or VC cells. **(F)** Apoptosis analysis by Annexin V/PI assay in MDA-MB-231 A6 and NTC cells cultured under an attached or detached condition for 0, 1 or 3 days. **(G)** Apoptosis analysis by Annexin V/PI assay in MCF7 CD147 or VC cells cultured under an attached or detached condition for three days. For immunoblot assay, α-tubulin was used as a loading control and one representative of three biological replicates was shown.

**Figure S4. CyPA-CD147 promotes CSC properties. (A)** Tumorsphere formation without or with passaging in MDA-MB-231 A6 and NTC cells (*left two*), or at the single cell level (*right*). **(B)** Tumor incidence and tumor growth in xenograft mouse models inoculated with MDA-MB-231 A6 cells and NTC cells. (**C)** Flow cytometry analysis of ALDH^+^ subpopulation in MDA-MB-231 A6 and NTC cells cultured under an attached or detached condition for three days. A representative of three biological replicates was shown.  **(D)** Immunoblot assay of OCT4 and SOX2 protein levels in MCF7, T-47D and MCF7 CD147/VC cells cultured under an Ad or AR condition for seven days. α-tubulin was used as a loading control and one representative of three biological replicates was shown. **(E)** ELISA analysis of supernatant CyPA levels from the conditioned medium of MDA-MB-231 cells cultured under an attached or detached condition for three days. **(F)** Flow cytometric analysis of ALDH^+^ subpopulation in MCF7 cells treated with 0, 1, 10 or 100 ng/mL CyPA for one day.

**Figure S5.** **CyPA increases CSC ratios *via* activating CD147.**  **(A-B)** Flow cytometric analysis of ALDH^+^ (A) or CD44^high^CD24^-/low^ (B) subpopulation in MDA-MB-231 A6 and NTC cells treated with or without 20 ng/mL CyPA for one day. **(C-D)** Flow cytometric analysis of ALDH^+^ (C) or CD44^high^CD24^-/low^ (D) subpopulation in T-47D CD147 and VC cells treated with or without 20 ng/mL CypA, 20 μmol/L CyPA inhibitor TMN 355 for one day.  **(E-F)** Flow cytometric analysis of ALDH^+^ (E) or CD44^high^CD24^-/low^ (F) subpopulation in MCF7 CD147 and VC cells treated with or without 20 ng/mL CypA, 20 μmol/L CyPA inhibitor TMN 355 for one day.

**Figure S6. CD147 and/or pSTAT3 expression is associated with poor overall survival or recurrence in breast cancer patients. (A)** STAT3, pSTAT3^Y705^ and Bcl-xL protein levels in MDA-MB-231 A6 and NTC cells cultured under an Ad or AR condition for seven days. α-tubulin was used as a loading control and one representative of three biological replicates was shown. **(B)** Kaplan-Meier curve analysis of overall survival and tumor recurrence time in breast cancer patients stratified by level of membranous CD147 and/or pSTAT3. ***Up:*** Overall survival (*left*) and tumor recurrence time (*right*) in 138 patients stratified by low- *vs.* high CD147 expression. ***Down:*** Overall survival (*left*) and tumor recurrence time (*right*) in 50 patients stratified by low- *vs.* high CD147/pSTAT3 expression. **(C)** Immunofluorescence staining of cytoplasmic CD147 and pSTAT3^Y705^ in breast cancer tissues specimens. The representative images were taken at magnification of ×20. Bar: 20 μM. **(D)** Immunostaining of cytoplasmic CD147 and pSTAT3^Y705^ in attatched or detached breast cancer cells. The representative images were taken at magnification of ×100. Bar: 10 μM. **(E)** Association of concurrent expression of cytoplasm CD147 (***up***) or membranous CD147 (***down***) with pSTAT3 in 138 breast cancer patients.
