# Supplementary material for: Detachment Activated CyPA/CD147 Induces Cancer Stem Cell Potential in Non-stem Breast Cancer Cells
Source: Front Cell Dev Biol. 2020 Oct 16;8:543856. doi: 10.3389/fcell.2020.543856 (PMC7640948; doi:10.3389/fcell.2020.543856)
Supplement: Supplementary file 3 [file Presentation_1.PPTX]

## Slide 1
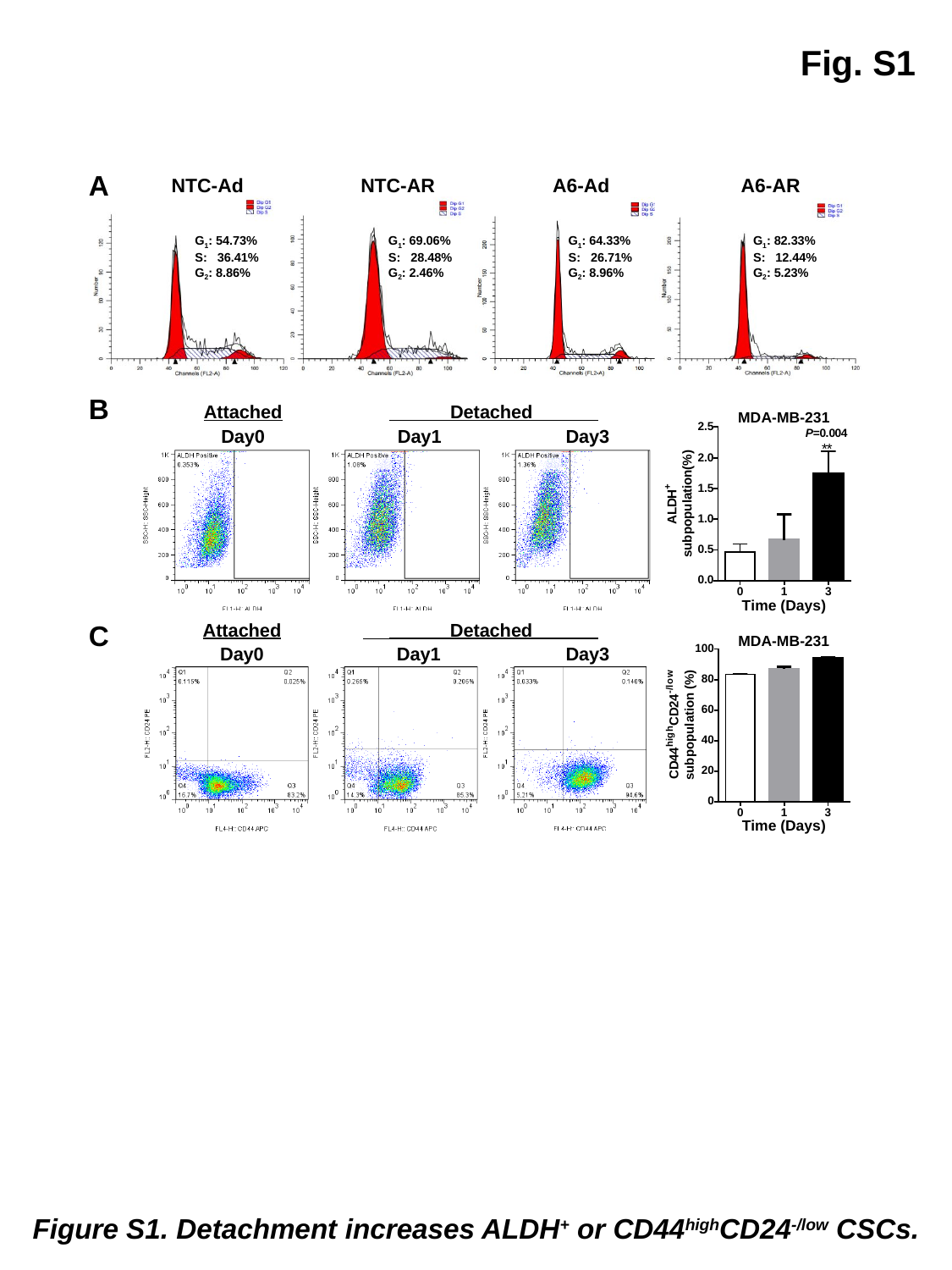

Fig. S1
A
NTC-Ad
NTC-AR
A6-Ad
A6-AR
G1: 54.73%
S: 36.41%
G2: 8.86%
G1: 69.06%
S: 28.48%
G2: 2.46%
G1: 64.33%
S: 26.71%
G2: 8.96%
G1: 82.33%
S: 12.44%
G2: 5.23%
B
Attached
 Detached
Day0
Day1
Day3
 Detached
Attached
Day0
Day1
Day3
C
Figure S1. Detachment increases ALDH+ or CD44highCD24-/low CSCs.

## Slide 2
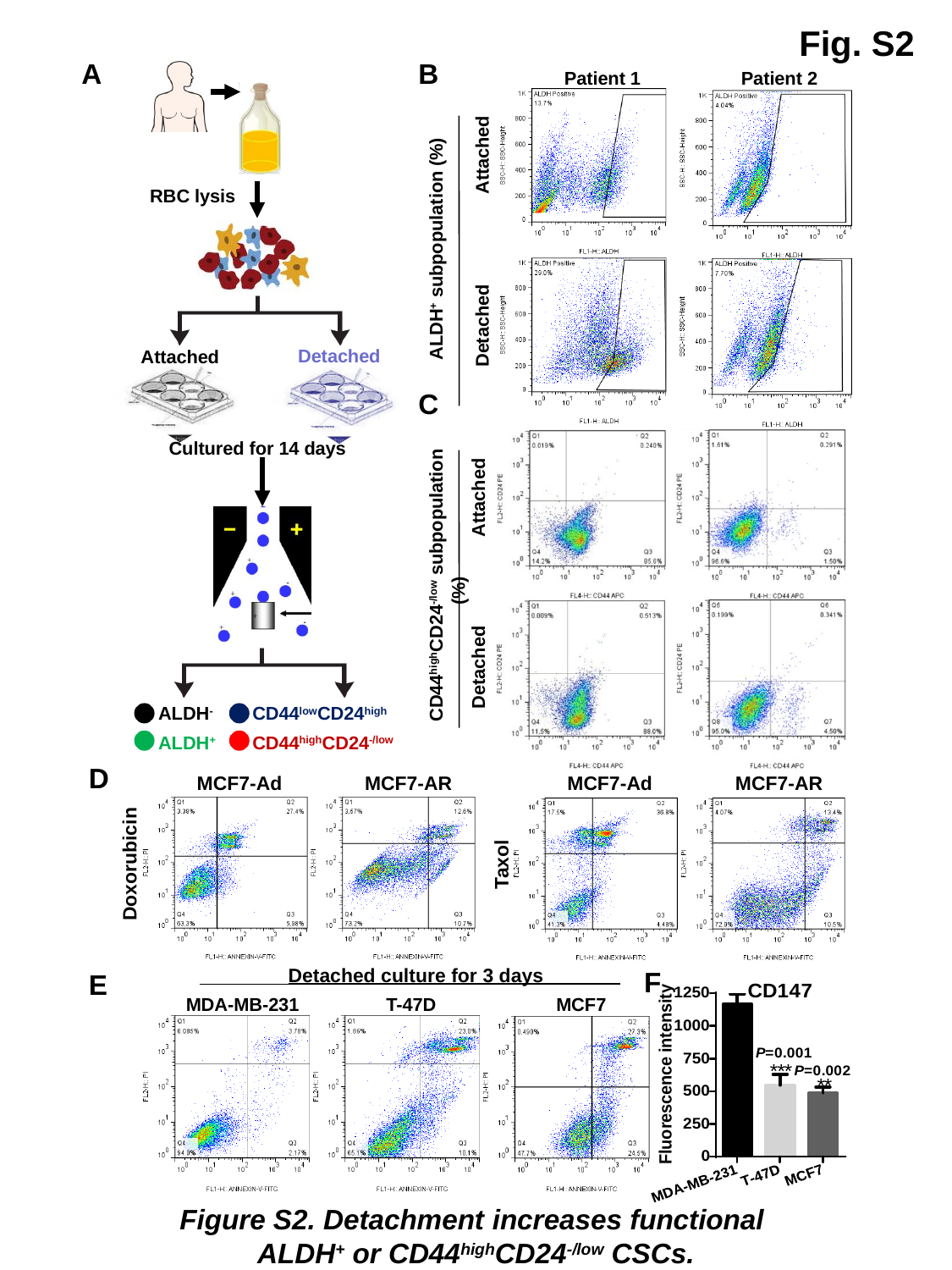

Fig. S2
A
B
RBC lysis
Detached
Attached
Cultured for 14 days
ALDH-
CD44lowCD24high
ALDH+
CD44highCD24-/low
Patient 1
Patient 2
 ALDH+ subpopulation (%)
Attached
Detached
 CD44highCD24-/low subpopulation (%)
Attached
Detached
C
D
MCF7-Ad
MCF7-AR
MCF7-Ad
MCF7-AR
Taxol
Doxorubicin
 Detached culture for 3 days
MDA-MB-231
T-47D
MCF7
F
E
Figure S2. Detachment increases functional
ALDH+ or CD44highCD24-/low CSCs.

## Slide 3
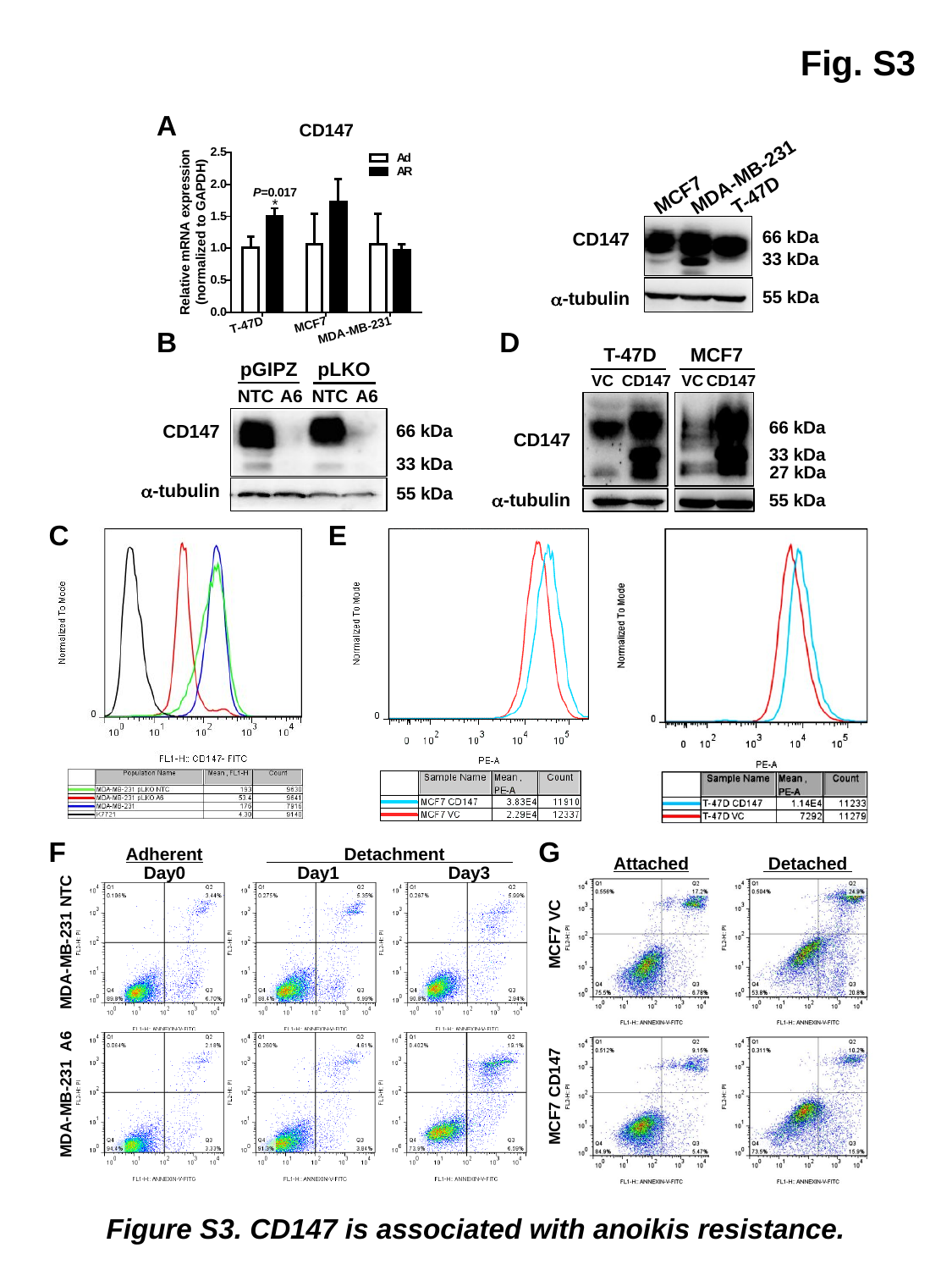

Fig. S3
A
MCF7
MDA-MB-231
T-47D
66 kDa
CD147
33 kDa
55 kDa
α-tubulin
B
D
MCF7
T-47D
VC
 CD147
VC
 CD147
66 kDa
CD147
33 kDa
α-tubulin
55 kDa
27 kDa
pGIPZ
pLKO
NTC
A6
NTC
A6
CD147
α-tubulin
66 kDa
33 kDa
55 kDa
C
E
F
G
Adherent
 Detachment
Day1
Day3
Day0
MDA-MB-231 NTC
MDA-MB-231 A6
Attached
 Detached
MCF7 VC
MCF7 CD147
Figure S3. CD147 is associated with anoikis resistance.

## Slide 4
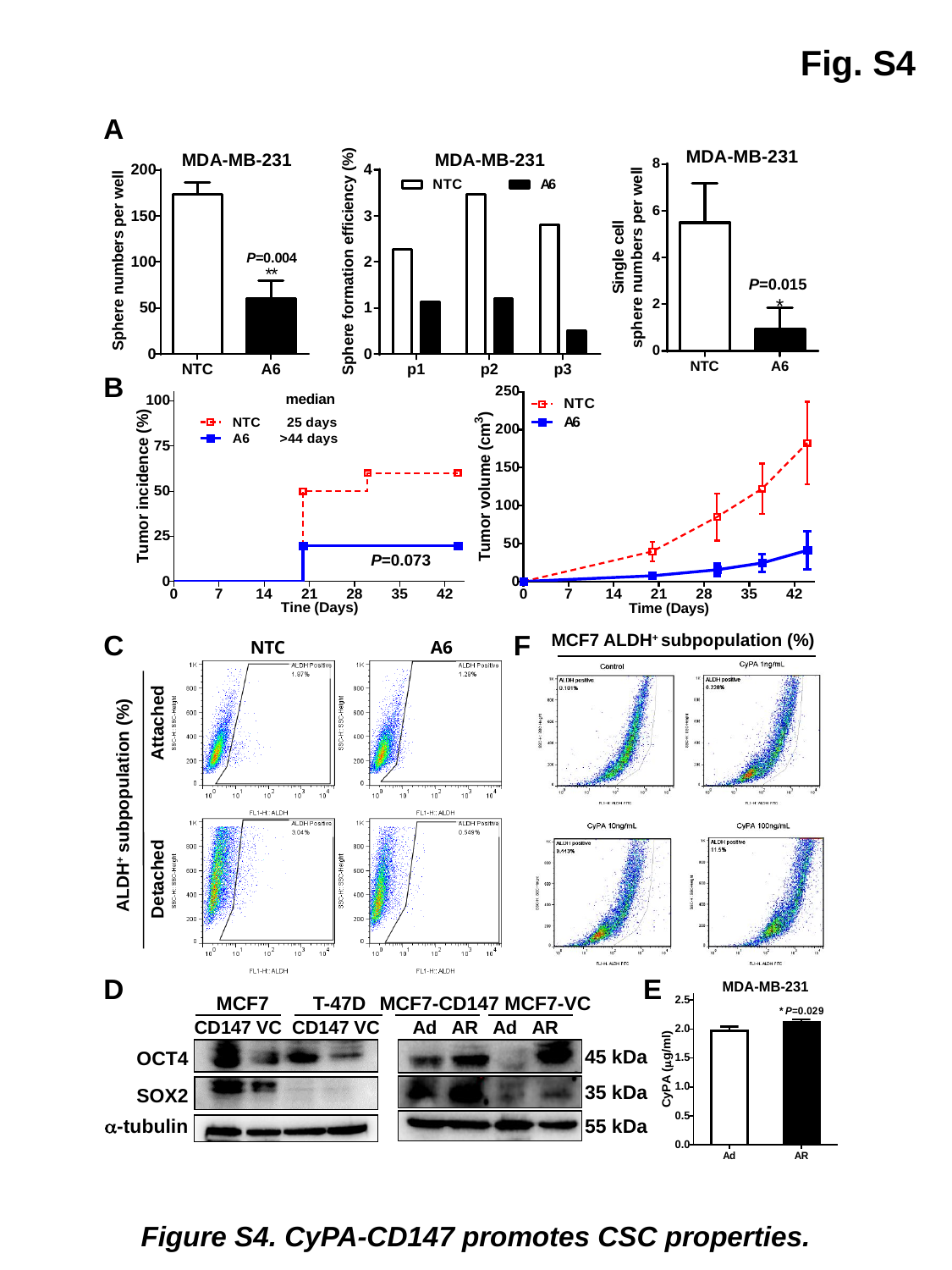

Fig. S4
A
B
C
F
MCF7 ALDH+ subpopulation (%)
　　　　NTC 　　　　　　　A6
ALDH+ subpopulation (%)
Attached
Detached
D
E
 MCF7 T-47D CD147 VC CD147 VC
 MCF7-CD147 MCF7-VC
 Ad AR Ad AR
45 kDa
OCT4
35 kDa
SOX2
α-tubulin
55 kDa
Figure S4. CyPA-CD147 promotes CSC properties.

## Slide 5
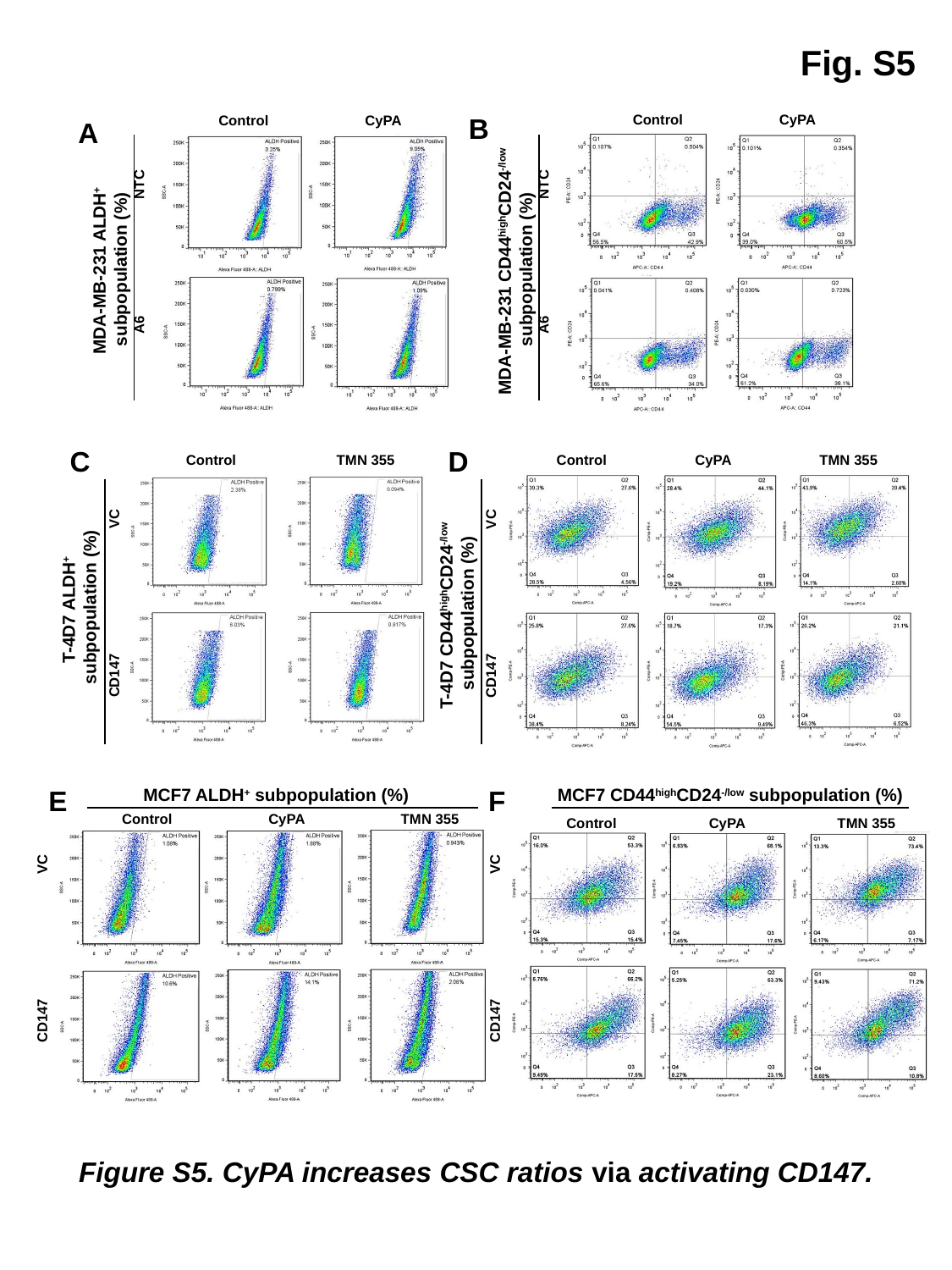

Fig. S5
Control CyPA
B
MDA-MB-231 CD44highCD24-/low
subpopulation (%)
A6 NTC
Control CyPA
A
A6 NTC
MDA-MB-231 ALDH+
subpopulation (%)
C
Control TMN 355
CD147 VC
T-4D7 ALDH+
subpopulation (%)
D
Control CyPA TMN 355
CD147 VC
T-4D7 CD44highCD24-/low
subpopulation (%)
E
MCF7 ALDH+ subpopulation (%)
Control CyPA TMN 355
CD147 VC
F
MCF7 CD44highCD24-/low subpopulation (%)
Control CyPA TMN 355
CD147 VC
Figure S5. CyPA increases CSC ratios via activating CD147.

## Slide 6
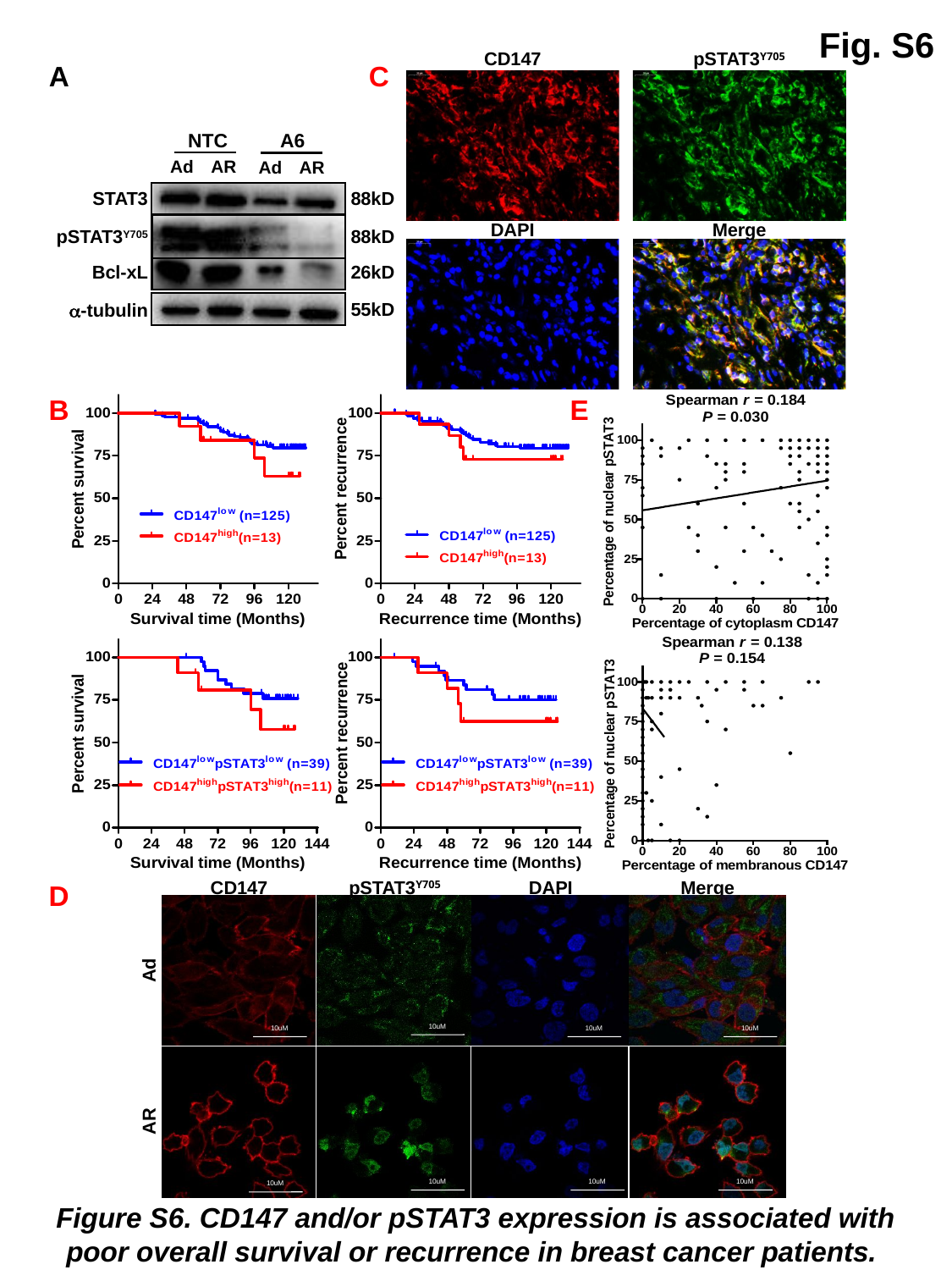

Fig. S6
CD147
pSTAT3Y705
DAPI
Merge
A
C
NTC
A6
Ad
AR
Ad
AR
STAT3
88kD
pSTAT3Y705
88kD
Bcl-xL
26kD
55kD
α-tubulin
B
E
CD147
pSTAT3Y705
DAPI
Merge
Ad
AR
D
10uM
10uM
10uM
10uM
10uM
10uM
10uM
10uM
Figure S6. CD147 and/or pSTAT3 expression is associated with poor overall survival or recurrence in breast cancer patients.
